# Alterations in the fecal microbiota of patients with spinal cord injury

**DOI:** 10.1371/journal.pone.0236470

**Published:** 2020-08-04

**Authors:** Ruizhu Lin, Jianfeng Xu, Qi Ma, Meihua Chen, Lei Wang, Sha Wen, Caixia Yang, Chuan Ma, Yue Wang, Qiang Luo, Ning Zhu

**Affiliations:** 1 Department of Rehabilitation Medicine, General Hospital of Ningxia Medical University, Yinchuan, Ningxia, China; 2 Second Department of Rehabilitation, the Designated Rehabilitation Cooperation Hospital of General Hospital of Ningxia Medical University, Yinchuan, Ningxia, China; 3 Traditional Chinese Medicine and Traumatology, Ningxia Medical University, Yinchuan, Ningxia, China; University of Maine, UNITED STATES

## Abstract

**Objectives:**

Spinal cord injury (SCI) is associated with severe autonomic dysfunction. Patients with SCI often suffer from a lack of central nervous system control over the gastrointestinal system. Therefore, we hypothesized that patients with SCI would cause intestinal flora imbalance. We investigated alterations in the fecal microbiome in a group of patients with SCI.

**Methods:**

Microbial communities in the feces of 23 patients and 23 healthy controls were investigated using high-throughput Illumina Miseq sequencing targeting the V3-V4 region of the 16S ribosomal RNA (rRNA) gene. The relative abundances between the fecal microbiota at the genus level in patients with SCI and healthy individuals were determined using cluster analysis.

**Results:**

The structure and quantity of fecal microbiota differed significantly between patients with SCI and healthy controls, but the richness and diversity were not significantly different. A two-dimensional heatmap showed that the relative abundances of forty-five operational taxonomic units (OTUs) were significantly enriched either in SCI or healthy samples. Among these, 18 OTUs were more abundant in healthy controls than in patients with SCI, and 27 OTUs were more abundant in the SCI group than in healthy controls.

**Conclusion:**

Our study showed that patients with SCI exhibited microbiome dysbiosis.

## 1. Introduction

Spinal cord injury (SCI) is a common central nervous system trauma in humans. Many patients with SCI suffer from severe neurological impairment, including loss of motor, sensory, and autonomic function, lumbar spine, femoral neck and shaft, and proximal tibia pain [1, 2], abdominal pain, abdominal distention, ileus, constipation, and other gastrointestinal disorders [3]. SCI generally results from accidents and falls, gunshot wounds, diving accidents, and other medical/surgical complications [4]. Increasing numbers of people suffer from SCI each year, with a worldwide incidence of 15 to 40 cases per million in 2001 [5]. In 2017, between 250,000 and 500,000 suffered from SCI worldwide [6]. In 2018, there were approximately 17,000 new cases of SCI [7].

SCI was considered a lifelong neurological disorder due to limited treatment options [8]. Increased understanding of the nature of SCI has led to development of new therapeutic strategies for treatment of SCI. Treatments such as cell transplantation, gene therapy, cytokine treatment, and use of biomaterial scaffolds have been widely used and studied, but have not resulted in full functional recovery [9]. However, spinal cord repair remains a significant goal of SCI therapeutic research. Targeting the gut and immune system in patients with SCI may have therapeutic value, as these systems are easier to target due to their sensitivities to changes in the gut microbiota. Previous studies have shown that changes or diversification in the composition of the intestinal microbiome were associated with several atopic diseases and obesity [10].

The gut microbiota has been likened to an organ comprised of prokaryotic cells with measurable functions, resulting in a unique intestinal ecosystem that functions symbiotically with host eukaryotic cells. Metagenomic and metabolomic technologies have shown that the gut microbiota contributes significantly to normal functioning of host organisms [11]. Neurological dysfunction may occur in up to 50% of spinal cord compression cases at injury onset [12, 13]. Central or peripheral nerves that control the intestinal tract are often injured during SCI, resulting in changes in intestinal microbiota [14]. In this study, 16S rRNA gene sequencing was used to analyze and compare the differences in microbiota communities in feces of patients with SCI and healthy controls.

## 2. Materials and methods

### 2.1 Patients and controls

All patients with SCI were recruited from the SCI clinic of General Hospital of Ningxia Medical University. The course of disease ranges from 8 to 14 months. Healthy subjects were recruited from the qualified population for physical examination of community residents in Yinchuan City (Ningxia, China). Written informed consent was obtained from each participant. Patients with SCI were not given antibiotics during the first three months of fecal specimen collection. This study was approved by the ethics committee of General Hospital of Ningxia Medical University (Approval No.: 2017–200).

### 2.2 Sample collection and DNA isolation

Forty-six fresh samples (23 patients with SCI and 23 healthy controls) were collected. Fresh samples of subjects were collected and placed in separate 20 ml sterile PV bottles by themselves in the morning, and sealed. Then immersed in liquid nitrogen and transferred to a freezer maintained at −80°C for cryopreservation in the laboratory. Complete the entire sampling process in 30 min. Total fecal DNA was extracted from stool samples using the Stool DNA Isolation Kit (FORE GENE, China) according to the instructions. Briefly, Lysozyme was used to break the wall of fecal bacteria for 10 min, and the supernatant was centrifuged. After that, the supernatant was centrifuged again for 5 min after enzymatic hydrolysis of the sample. The impurities were then removed, DNA was absorbed by DNA adsorption column, and the impurities were washed with ultrapure water and then the DNA was eluted.

### 2.3 16SrRNA gene amplification

After extracting fecal genomic DNA, the V3–V4 regions of the 16S rRNA gene were amplified using PCR (95°C for 3 min, followed by 21 cycles at 94°C for 30 s, 58°C for 30 s, 72°C for 30 s, then 72°C for 5 min) with the universal primers 338F 5′-ACTCCTACGGGAGGCAGCA-3′ and 806R 5′-GGACTACHVGGGTWTCTAAT-3′. After amplification, PCR products were separated on a 2% agarose gel to confirm amplification success. A total of 4,444,553 reads were sequenced and obtained using an Illumina Miseq platform.

### 2.4 Bioinformatics and statistical data analyses

R software (ver. 3.1.0) was used for statistical analysis. Student’s t-test and Fisher exact test were used to compare quantitative and categorical variables, respectively, between the SCI group and the healthy group. Significant differences in the microbiota at the genus-level were determined using the Wilcoxon rank-sum test, and the microbiota that could determine the occurrence of SCI were predicted using the random forest (RF) model with default parameters in the "randomForest" package [15]. The accuracy of this model was evaluated by generating a receiver operating characteristic (ROC) curve and calculating the area under the ROC curve (AUC). During the clustering process, the chimera was removed to obtain the OTU representative sequence, and then OTU clustering was performed on the non-repetitive sequence (without single sequence) according to 97% similarity. In order to obtain the species classification information corresponding to each OTU, the representative sequences were compared with silva database to obtain the species information contained in each sample, and the RDP classifier Bayesian algorithm was used to conduct taxonomic analysis on 97% similar level OTU representative sequences. All analyses related to intestinal microbial communities were based on the relative abundances of OTUs in each sample, and the Bray–Curtis index was used as a distance measure. A two-dimensional heatmap was generated to show the relative abundance of microbial indicator OTUs between the SCI and healthy samples using the vegan package [16]. P < 0.05 was defined as significantly different.

## 3. Results

### 3.1 Patients and control groups

The basic characteristics of patients with SCI and healthy controls are summarized in Table 1. Twenty-three patients (nineteen males and four females) with SCI were included in the experimental group. These patients suffered from differing degrees of SCI as follows: 5 patients had complete SCI and 18 patients had incomplete SCI. The positions of the spinal cord injuries were as follows: 3 cases of cervical injury, 12 cases of thoracic injury, and 8 cases of lumbar injury. In addition, 23 healthy subjects (fifteen males and eight females) with no history of SCI were included in the control group. None of the subjects in either group suffered from any additional diseases or disorders.

**Table 1:** Characteristics of patients with SCI and healthy controls.

Variable	Patients with SCI (n = 23)	Healthy controls (n = 23)
Gender (male; female)	19; 4	15; 8
Age (mean ± standard)	32 ± 2.23	28 ± 3.45
Months from injury (mean ± standard)	11 ± 2.68	-
Injury position	-	-
Cervical segment	3	-
Thoracic segment	12	-
Lumbar segment	8	-
Injury degree	-	-
Complete	5	-
Incomplete	18	
Diabetes	-	-
High blood pressure	-	-
Coronary heart disease	-	-

“-”: Not applicable.

### 3.2 Alpha and beta diversity

**Fig 1:**
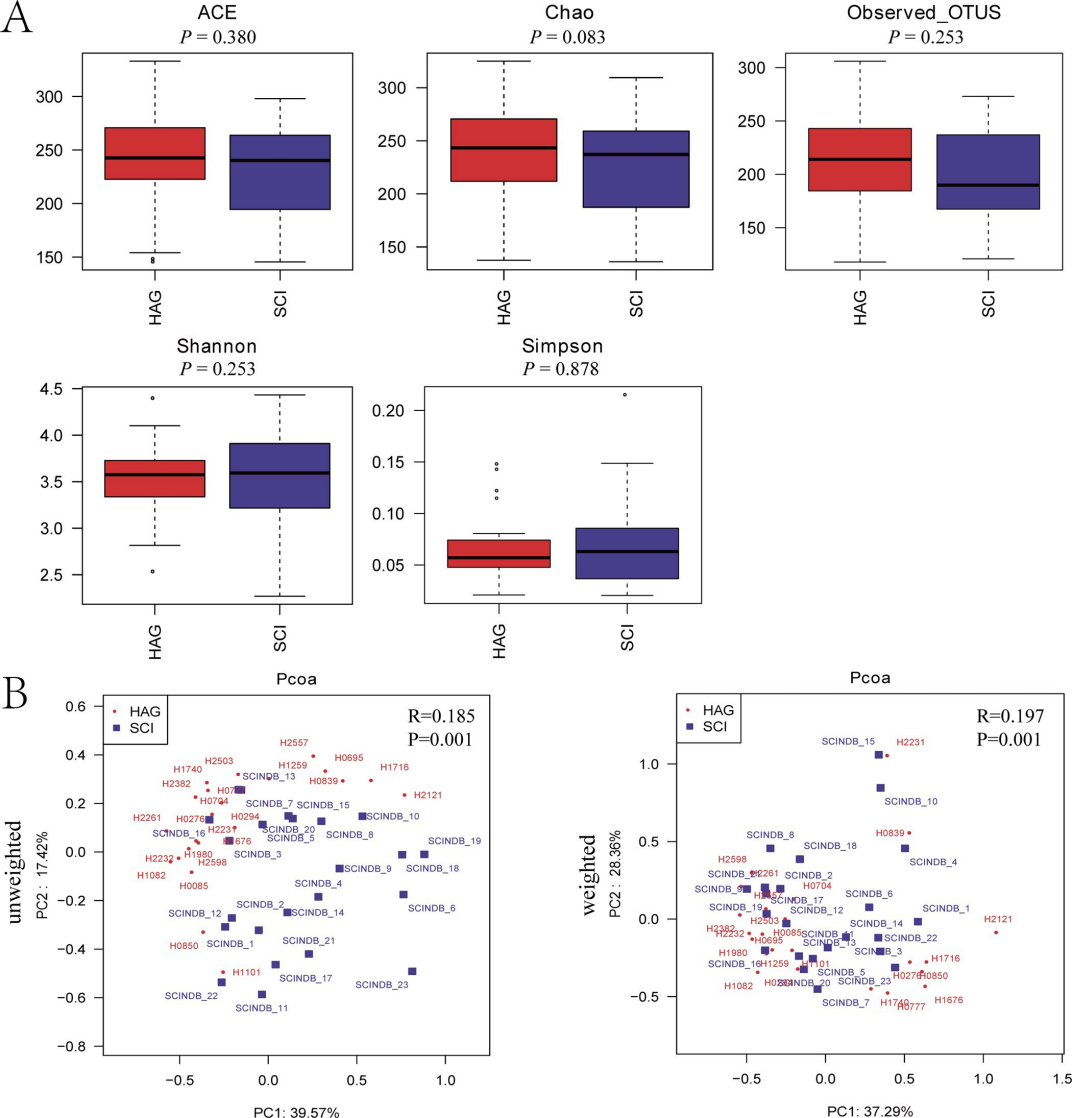
The α-diversity and β-diversity indices of the fecal microbiome in the SCI and healthy groups. (A) Box plots showing differences in the fecal microbiome diversity indices between the SCI and healthy groups according to the ACE, Chao, Observed_OTUs, Shannon, and Simpson diversity indices based on OTU levels. Each box plot represents the median, interquartile range, minimum, and maximum values. (B) Unweighted and weighted ANOSIMs and PCoA based on the UniFrac distance matrix of fecal microbial communities in the SCI and healthy groups. Respective ANOSIM R values show the community differences between the compared groups. The axes represent the two dimensions explaining the greatest ratio of variance in the communities. Each symbol represents a sample. SCI, spinal cord injury group (blue); HAG, healthy group (red); OTU, operational taxonomic unit; ANOSIM, analyses of similarities; PCoA, principal coordinates analysis.

The α-diversity, as determined by ACE, Chao, Observed OTUs, Shannon, and Simpson, based on OTU levels in the fecal microbiota, was determined in patients with SCI and healthy controls. No significant differences were observed in the α-diversity indices (richness and diversity) of the fecal microbiota between the SCI and control groups. The ACE, Chao, and OTU indices were slightly lower in the SCI group than those in the control group. In contrast, the Shannon and Simpson indices were slightly higher in the SCI group than those in the control group (Fig 1A, P > 0.05). Significant differences were observed in β-diversity based on the unweighted (qualitative, analysis of similarities [ANOSIM] R = 0.185, P = 0.001) and the weighted (quantitative, ANOSIM R = 0.197, P = 0.001) UniFrac between the SCI and control groups (Fig 1B, P < 0.05).

### 3.3 Taxonomic differences

**Fig 2:**
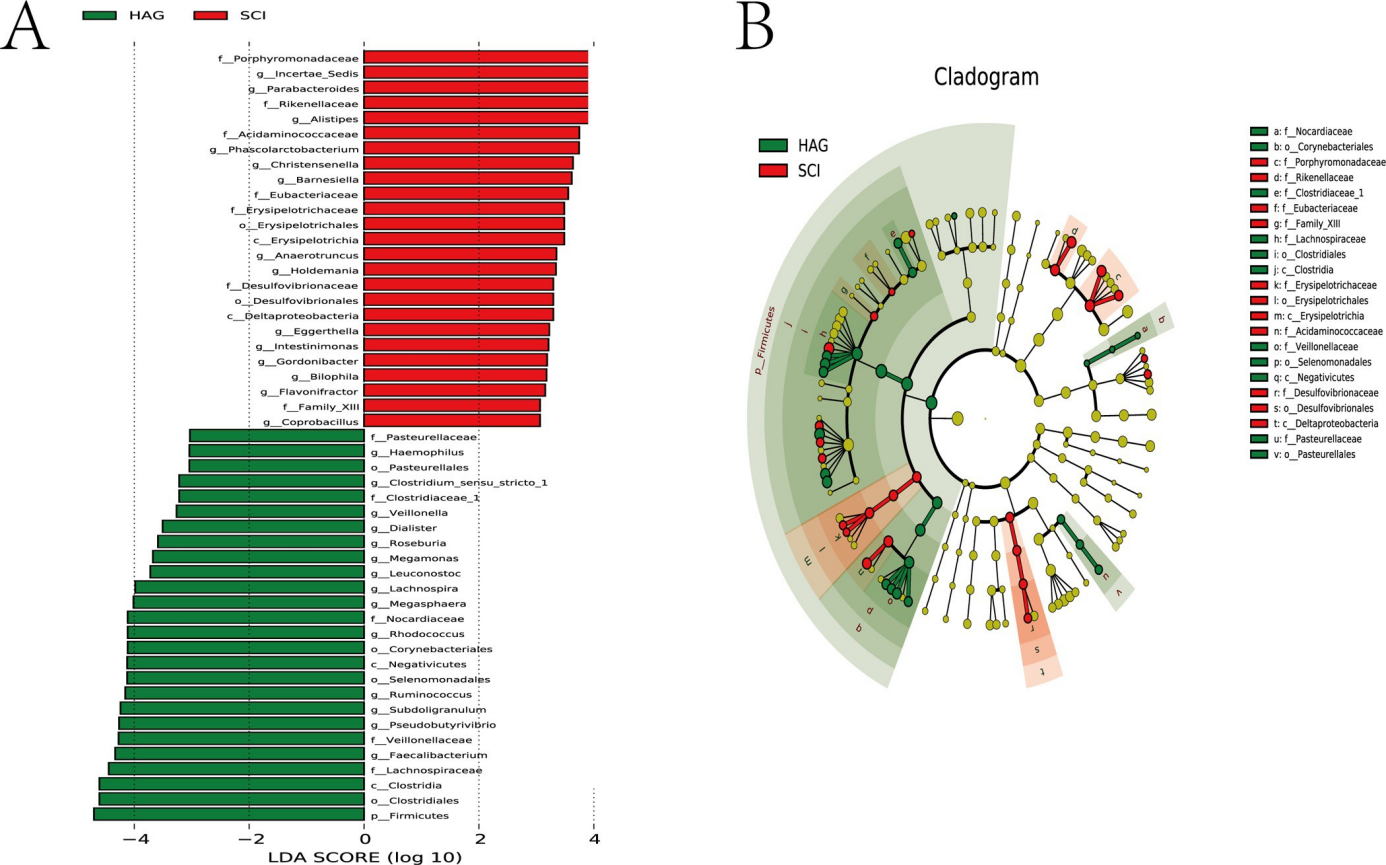
Taxonomic differences of fecal microbiota in the SCI and healthy groups. (A) Linear discriminant analysis (LDA) effect size (LEfSe) analysis showed significant differences in bacterial abundances in the fecal microbiota between the SCI (positive score) and healthy groups (negative score). Linear discriminant analysis scores (log10) > 2 and P < 0.05 are listed. (B) The LEfSe method was used to build a cladogram to analyze the phylogenetic distribution of fecal microbiota associated with patients with SCI and healthy subjects.

Linear discriminant analysis (LDA) effect size (LEfSe) was used to identify the communities or species that significantly contributed to differences between the SCI and the control group, and to determine differences in region-specific OTUs [17]. A logarithmic LDA score cutoff of 2.0 and P value < 0.05 was used to distinguish important taxonomic differences between these two groups. Significant differences in genera taxa were observed in the fecal microbiota between the SCI and the control group. The relative abundances of the genera *Parabacteroides*, *Alistipes*, *Phascolarctobacterium*, *Christensenella*, *Barnesiella*, *Holdemania*, *Eggerthella*, *Intestinimonas*, *Gordonibacter*, *Bilophila*, *Flavonifractor*, and *Coprobacillus* were higher in the SCI group than those in the control group. In contrast, the relative abundances of the genera *Haemophilus*, *Clostrdium sensu stricto* 1, *Veillonella*, *Dialister*, *Roseburia*, *Megamonus*, *Leuconostoc*, *Lachnospira*, *Megasphaera*, *Rhodococcus*, *Ruminococcus*, *Subdoligranulum*, *pesudobeautyrivibrio*, and *Faecalibacterium* were higher in the control group than those in the SCI group. (LDA score (log10) > 2, Fig 2A and 2B).

### 3.4 Random Forest (RF) predictive model

**Fig 3:**
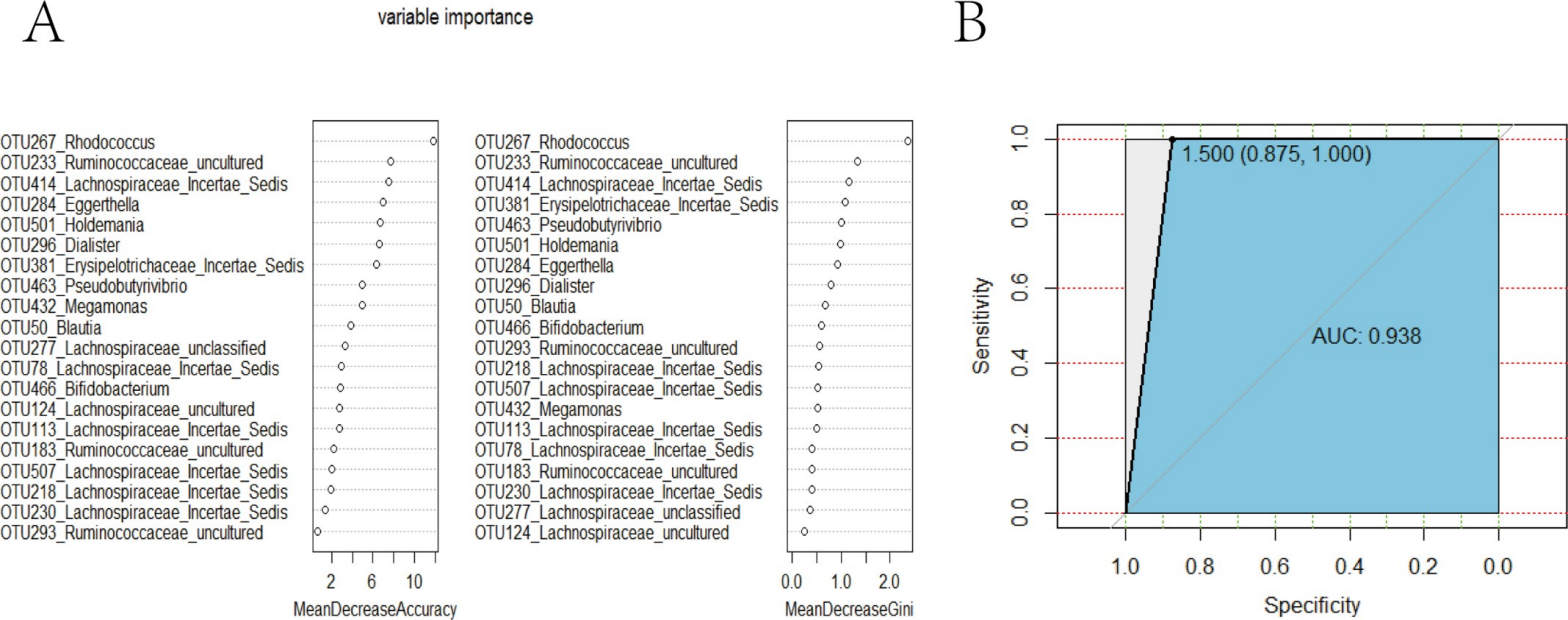
Decision trees to evaluate the fecal microbiota after SCI. (A) An RF predictive model based on genus-level abundance taxa was used to predict the relative importance of each genus based on the mean decreasing accuracy and the Gini coefficient for fecal microbiota. (B) An ROC curve generated using the RF analysis of 20 genera in the fecal microbiota. The ROC curve shows the corresponding optimal threshold. RF, Random Forest; ROC, receiver operating characteristic; AUC, area under the ROC curve.

Independent of healthy controls, a predictive model consisting of 20 relatively important genera was identified, which may predict the presence of SCI (Fig 3A). The area under the receiver operating characteristic curve (AUC) was 0.938, which indicated that the model had excellent predictive capability (Fig 3B, sensitivity 100% and specificity 87.5%).

### 3.5 Relative percent abundance of each genus

**Fig 4:**
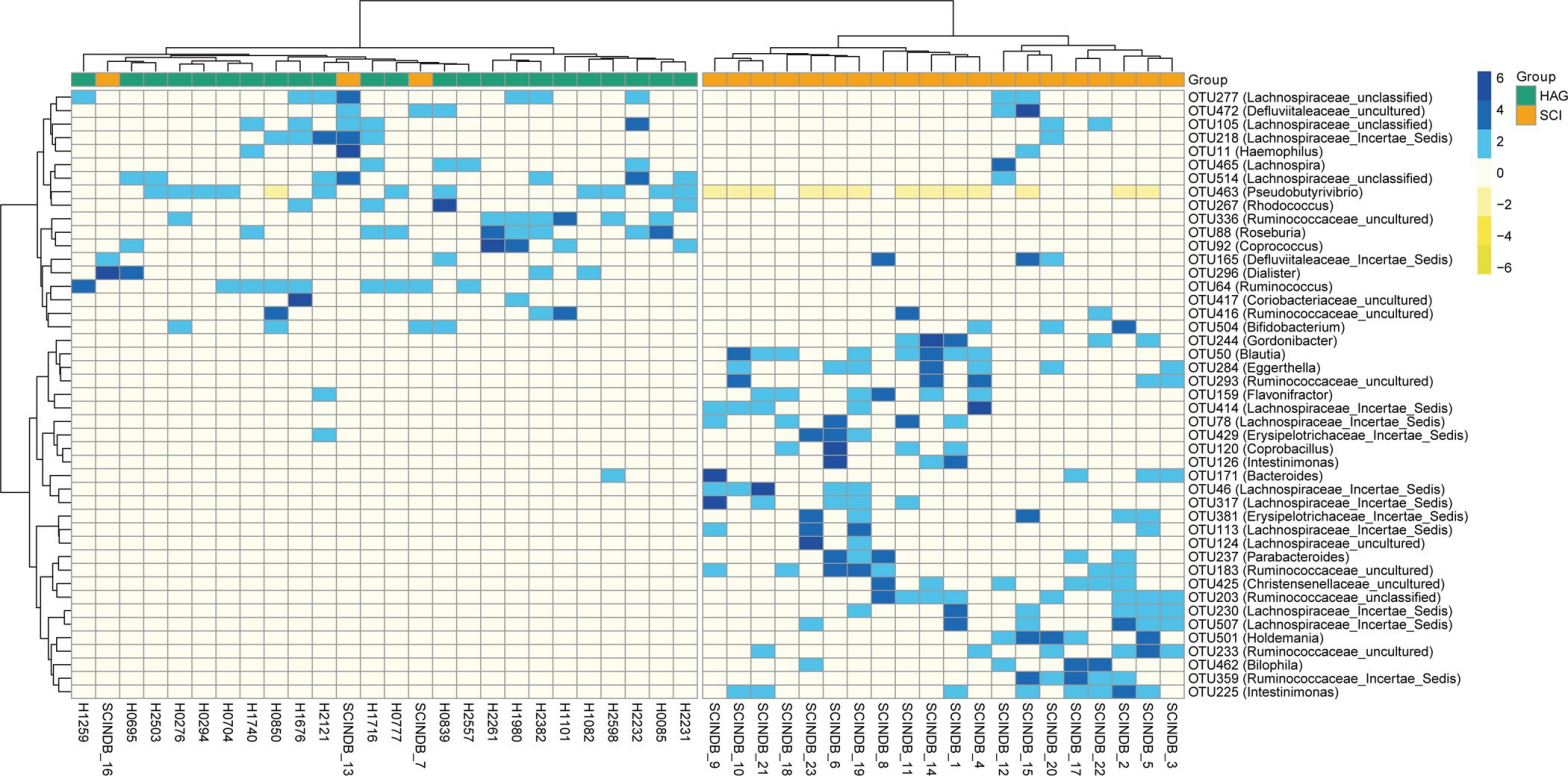
A two-dimensional heatmap showing the relative abundance of 45 dominant genera in 23 patients with SCI and 23 healthy controls. Rows represent the 45 bacterial genera, which were significantly enriched either in SCI or healthy samples, columns represent the 46 samples of SCI or healthy samples. The upper 18 OTUs exhibited higher relative abundance in the healthy group, and the lower 27 OTUs exhibited higher relative abundance in the SCI group. OTUs, operational taxonomic units. SCI, spinal cord injury group (orange); HAG, healthy group (green).

To determine whether SCI altered the overall composition of the intestinal microbial community at the genus level, a two-dimensional heatmap of the 45 most dominant genera is shown in Fig 4, which further illustrates the distinct patterns of gut bacterial composition in SCI and healthy samples. A hierarchical clustering based on relative abundances of different genera could sufficiently segregate SCI and healthy samples. On the other hand, two distinct groups of bacterial genera could be identified, which exhibited contrasting abundance trends in SCI and healthy samples. Of these, 18 OTUs were more abundant in control subjects than in patients with SCI, and 27 OTUs were significantly more abundant in the SCI group than in the control group. Overall, the composition of intestinal microorganisms was significantly different between the SCI group and the control group (Fig 4, P < 0.05).

## 4. Discussion

In this study, we hypothesized that the intestinal microbiota of patients with SCI was significantly different from that of healthy controls. Thus, we tested our hypothesis in 23 patients with SCI and 23 healthy controls using 16S rRNA sequencing. The patient and control groups were age-matched and had not received antibiotics within 3 months of the study. The results of this study showed that patients with SCI had significantly different intestinal microbiota than controls, which may explain the possible link between gut microbiota disorder and SCI.

Previous studies have linked dysregulation of gut microbiota to SCI. Kigerl *et al*. demonstrated that gut dysbiosis occurs with SCI in mice, and premorbid dysbiosis leads to poorer neurological outcomes and neuropathological findings post-injury [18]. Gungor *et al*. conducted a clinical study that included 30 patients with SCI and 10 healthy controls, and found that some genera, such as *Roseburia*, *Pseudobutyrivibrio*, *Dialister*, and *Megamonas*, which are butyrate-producing bacteria, were significantly less abundant in patients with SCI than in healthy subjects [14]. Zhang *et al*. showed that the diversity of the gut microbiota was altered in 43 adult patients with chronic complete SCI, and also showed that gut microbiota dysbiosis in patients with SCI was associated with neurogenic bowel dysfunction (NBD) symptoms [19]. A mouse (C57BL/6, females) study showed that *Bacteroidales* and *Clostridiales*, the two major bacterial orders in the gut, were regulated by SCI, which suggested that the gut microbiota may be associated with the pathogenesis of SCI [18]. These findings highlighted the importance of the gut microbiota in patients with SCI in the post-injury phase.

In the present study, no significant differences were observed in α-diversity indices (richness and diversity) of the fecal microbiota between the SCI and control groups. However, significant differences in β-diversity indices (structure and quantity) were identified between the two groups, which indicated that the composition of fecal microbial in the SCI group was distinctly different from that of the healthy group. In previous studies, Zhang *et al*. reported that patients with SCI and healthy controls showed significant differences in α-diversity and β-diversity indices in the gut microbiota. The differences in α-diversity were characterized by increased community richness and decreased community diversity in patients with SCI. These results were not consistent with our findings [19].

We used 16S rRNA gene sequence informatics to perform genus identification and to determine specific differences at the genus level. Significant bacterial differences in fecal microbiota were associated with SCI. Additionally, in our study, we also established a predictive model comprised of 20 relatively important OTUs with excellent predictive value. Furthermore, we determined the relative abundances of these OTUs in the fecal microbiota of patients with SCI and healthy subjects. The composition of 45 intestinal microorganisms was significantly different at the genus level, based on analysis using the Wilcoxon rank-sum test. Among these, *Rhodococcus* (OTU267), *Dialister* (OTU296), *Pseudobutyrivibrio* (OTU463) were decreased, *Lachnospiraceae Incertae Sedis* (OTU414), *Eggerthella* (OTU284) were increased in SCI groups. Interestingly, these changes were also observed in previous studies. For example, *Eggerthella *was isolated in tissue cultures from an 82-year-old Chinese woman with spondylodiscitis that presented with low back pain for two weeks [20]. The relative abundance of the family *Lachnospiraceae* was increased in rats with femur fracture, limb skeletal muscle and small intestine without resuscitation [21]. A pure culture of *Rhodococcus* was also isolated from the right occipitoatlantal joint and cerebrospinal fluid of a 3-month-old female thoroughbred foal with cranial cervical SCI, occipital bone osteomyelitis, and atlanto-occipital septic arthritis [22]. *Rhodococcus* was not present in the intestinal microbiota of the 23 patients with SCI in our study, which may be a significant finding. In these studies, SCI were associated with dysregulation of intestinal microbiota. Furthermore, these studies indicated that the abundances of *Eggerthella*, *Lachnospiraceae*, and *Rhodococcus* were affected by neurological dysfunction following SCI in our present study, and that these genera may be universal indicators of the intestinal consequences of SCI. *Dialister*, a member of *Bacteroides*, ferment indigestible polysaccharides and produce metabolites [23]. *Pseudobutyrivibrio* is likely to produce butyrate [24]. Gungor *et al*. showed significant differences among these two intestinal microbes between the SCI and control groups [14]. Interestingly, this study also revealed that low butyrate levels may affect the long-term recovery after SCI. Thus, our results indicated that the reduction of *Pseudobutyrivibrio* and *Dialister* in patients with SCI may impact long-term recovery and energy supply following SCI. In addition, other important intestinal bacteria that have not been previously linked to SCI were also significantly altered in our study. Thousands of intestinal microorganisms colonize the intestinal tract, and these microbiota and the products of their metabolic processes have significant effects on the host, such as regulation of the immune system [25], digestive system [26], and nervous system [27]. These results indicated that intestinal microbiota was significantly affected by SCI, which may be closely related to injury, infection, or neurological dysfunction following SCI. However, there were no significant difference in intestinal microbiota between incomplete and complete lesions, as well as cervical segment, thoracic segment, lumbar segment, which may be due to the small number of samples we collected (S1 Table).

The composition of the intestinal microbiome varies significantly among populations and individuals based on race, geography, host genes, age, and other factors, which has led to diverse results in studies of spinal cord injuries and intestinal microbiota disorders [28]. In our study, we determined the relative abundances of intestinal microbiota, and also determined changes in the gut microbiota at the genus level in patients with SCI based on gender, age, injury degree, and injury location, these results showed a clear connection between SCI and intestinal microbiota disorder. At present, more and more studies have found that the damage of some intestinal microbiota and its metabolite composition can also be used as a biomarker for the diagnosis of the progression of disease damage. Therefore, gut microbiota transplantation is committed to be used as potential therapeutic targets to improve disease dysfunction and reverse pathological conditions in the future. However, our study suffered from some limitations. First, the sample size was small, and larger studies are needed to confirm our experimental results. Second, 16S rRNA gene sequencing cannot directly identify functional changes in microbiota. Shotgun metagenomic analysis is needed to evaluate functional differences. Finally, individual differences in diet may have biased our results.

Overall, our findings showed dysbiosis of the gut microbiome in a cohort of patients with SCI, which will provide a scaffold for further studies to understand the specific links between intestinal microbiota and disease. However, due to the complexity and diversity of human intestinal microbiota, comparative studies between healthy subjects and patients with diseases do not adequately characterize how intestinal microbiota may cause intestinal dysfunction. Therefore, comprehensive studies on the mechanisms by which intestinal microbiota contribute to disease may allow for development of oral probiotics as therapeutic options.

## Supporting information

S1 File Combined data with relative percentages among groups. SCI: Spinal cord injury group (Group A, N = 23); H: Healthy controls (Group B, N = 23).
(ZIP)

S1 Table Serial number of patients with Spinal Cord Injury (SCI). (DOCX)
