# Supplementary material for: Alterations in the fecal microbiota of patients with spinal cord injury
Source: PLoS One. 2020 Aug 4;15(8):e0236470. doi: 10.1371/journal.pone.0236470 (PMC7402510; doi:10.1371/journal.pone.0236470)
Supplement: S1 File — SCI: Spinal cord injury group (Group A, N = 23); H: Healthy controls (Group B, N = 23). (ZIP) [file pone.0236470.s001.zip › Supporting Information/Sample introduction.docx]

SCI: Spinal cord injury group (Group A, N = 23); H: Healthy controls (Group B, N = 23).

| **Samples** | |
| --- | --- |
| Group A | H0085 |
|  | H0276 |
|  | H0294 |
|  | H0695 |
|  | H0704 |
|  | H0777 |
|  | H0839 |
|  | H0850 |
|  | H1082 |
|  | H1101 |
|  | H1259 |
|  | H1676 |
|  | H1716 |
|  | H1740 |
|  | H1980 |
|  | H2121 |
|  | H2231 |
|  | H2232 |
|  | H2261 |
|  | H2382 |
|  | H2503 |
|  | H2557 |
|  | H2598 |
| Group B | SCINDB_1 |
|  | SCINDB_2 |
|  | SCINDB_4 |
|  | SCINDB_5 |
|  | SCINDB_8 |
|  | SCINDB_9 |
|  | SCINDB_13 |
|  | SCINDB_14 |
|  | SCINDB_16 |
|  | SCINDB_17 |
|  | SCINDB_18 |
|  | SCINDB_3 |
|  | SCINDB_6 |
|  | SCINDB_7 |
|  | SCINDB_10 |
|  | SCINDB_11 |
|  | SCINDB_12 |
|  | SCINDB_15 |
|  | SCINDB_19 |
|  | SCINDB_20 |
|  | SCINDB_21 |
|  | SCINDB_22 |
|  | SCINDB_23 |
