# Supplementary material for: Alterations in the fecal microbiota of patients with spinal cord injury
Source: PLoS One. 2020 Aug 4;15(8):e0236470. doi: 10.1371/journal.pone.0236470 (PMC7402510; doi:10.1371/journal.pone.0236470)
Supplement: S1 Table — (DOCX) [file pone.0236470.s002.docx]

**S1 Table. Serial number of patients with spinal cord injury (SCI)**

|  | **Patients with SCI (n=23)** |
| --- | --- |
| Injury position | - |
| Cervical segment | 3 (SCINDB_3、SCINDB_5、SCINDB_11) |
| Thoracic segment | 12 (SCINDB_ 1、SCINDB_4、SCINDB_6、SCINDB_7、SCINDB_8、SCINDB_10、SCINDB_12、SCINDB_14、SCINDB_16、SCINDB_17、SCINDB_18、SCINDB_22) |
| Lumbar segment | 8 (SCINDB_2、SCINDB_9、SCINDB_13、SCINDB_15、SCINDB_19、SCINDB_20、SCINDB_21、SCINDB_23) |
| Injury degree | - |
| Complete | 5 (SCINDB_3、SCINDB_5、SCINDB_9、SCINDB_11、SCINDB_20) |
| Incomplete | 18 (SCINDB_1、SCINDB_2、SCINDB_4、SCINDB_6、SCINDB_7、SCINDB_8、SCINDB_10、SCINDB_12、SCINDB_13、SCINDB_14、SCINDB_15、SCINDB_16、SCINDB_17、SCINDB_18、SCINDB_19、SCINDB_21、SCINDB_22、SCINDB_23) |

“-”: Not applicable. Twenty-three SCI patients were grouped according to different injury locations and injury degrees, and the serial numbers were marked in the above table, respectively. The spinal levels of complete SCI and incomplete SCI in the present study are distinguished on the basis of these three (cervical, thoracic and lumbar) vertebral levels.
